# Targeting the Proline–Glutamine–Asparagine–Arginine Metabolic Axis in Amino Acid Starvation Cancer Therapy

**DOI:** 10.3390/ph14010072

**Published:** 2021-01-18

**Authors:** Macus Tien Kuo, Helen H. W. Chen, Lynn G. Feun, Niramol Savaraj

**Affiliations:** 1 Department of Translational Molecular Pathology, The University of Texas MD Anderson Cancer Center, Houston, TX 77030, USA; 2 Department of Radiation Oncology, National Cheng Kung University Hospital, College of Medicine, National Cheng Kung University, Tainan 70428, Taiwan; Helen@mail.ncku.edu.tw; 3 Department of Medicine, Sylvester Comprehensive Cancer Center, Miller School of Medicine, University of Miami, Miami, FL 33136, USA; lfeun@med.miami.edu; 4 Division of Hematology and Oncology, Miami Veterans Affairs Heaithcare System, Miami, FL 33136, USA; nsavaraj@med.miami.edu

**Keywords:** amino acid starvation therapy, proline, glutamine, arginine, asparagine, drug resistance

## Abstract

Proline, glutamine, asparagine, and arginine are conditionally non-essential amino acids that can be produced in our body. However, they are essential for the growth of highly proliferative cells such as cancers. Many cancers express reduced levels of these amino acids and thus require import from the environment. Meanwhile, the biosynthesis of these amino acids is inter-connected but can be intervened individually through the inhibition of key enzymes of the biosynthesis of these amino acids, resulting in amino acid starvation and cell death. Amino acid starvation strategies have been in various stages of clinical applications. Targeting asparagine using asparaginase has been approved for treating acute lymphoblastic leukemia. Targeting glutamine and arginine starvations are in various stages of clinical trials, and targeting proline starvation is in preclinical development. The most important obstacle of these therapies is drug resistance, which is mostly due to reactivation of the key enzymes involved in biosynthesis of the targeted amino acids and reprogramming of compensatory survival pathways via transcriptional, epigenetic, and post-translational mechanisms. Here, we review the interactive regulatory mechanisms that control cellular levels of these amino acids for amino acid starvation therapy and how drug resistance is evolved underlying treatment failure.

## 1. Introduction

Amino acid starvation therapy has been emerging as an important treatment strategy in cancer therapy. This strategy is based on the promise of differential requirements of amino acids for supporting cell proliferation between cancer cells and normal cells. The non-essential amino acids such as proline (Pro), glutamine (Gln), asparagine (Asn), and arginine (Arg) support this promise. While these amino acids can be synthesized endogenously in normal cells, many human tumors, ranging from leukemia to solid cancers, do not produce sufficient amounts of these amino acids in supporting their growth. This is because the key enzymes involved in biosynthetic pathways of these amino acids are silenced. This forces tumor cells to acquire extracellular sources of amino acids to support their intracellular need. Depleting these extracellular supplies result in amino acid starvation and cell death.

## 2. The Interconnecting Proline–Glutamine–Asparagine–Arginine Metabolic Wiring in Cancer Cells

Cells obtain amino acids from two major routes: one from the extracellular environment through various amino acid transporters [1] and the other from *de novo* biosynthesis. At least 32 human solute carriers (SLC), belonging to seven families, are involved in transporting amino acids. Many of them transport multiple amino acids; likewise, multiple amino acids can be transported by the same SLC. The high redundancies of these transporters in conjunction of interconnecting de novo biosynthetic processes of amino acids such as Pro, Gln, Asn, and Arg provide opportunities but also challenges for successful targeted amino acid starvation therapy that will be discussed here.

**Figure 1:**
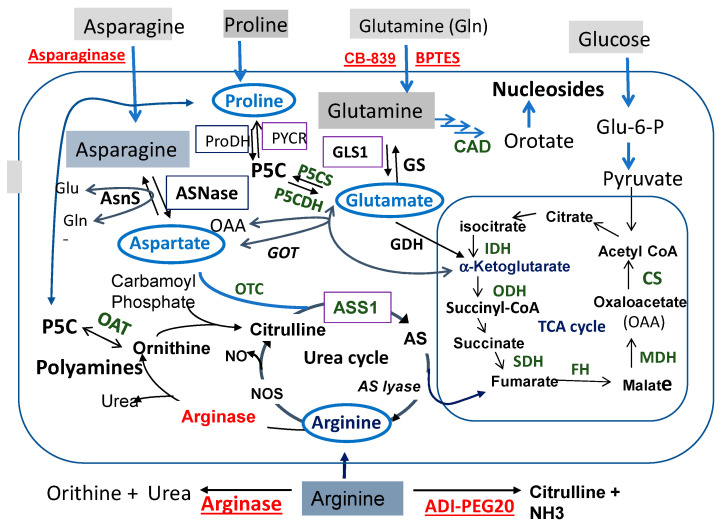
Metabolic pathways linking proline (Pro), glutamine (Glu), arginine (Arg), and asparagine (Asn). Abbreviations: AS, argininosuccinate; ASNase, asparaginase; AsnS, asparagine synthetase; ASS1; argininosuccinate synthetase 1; GDH, glutamine dehydrogenase; glutamic-oxaloacetic transaminase 1; FH, fumarate hydratase; GLS, glutaminase; GS, glutamine synthetase; GOT, glutamic oxaloacetic transaminase 1; GDH, glutamine dehydrogenase; NOS, nitric oxide synthetase; OAA, oxaloacetate; OAT, Ornithine aminotransferase; OTC, ornithine transcarbamylase; P5C, pyrroline 5-carboxylate; ProDH, proline dehydrogenase; PYCR, P5C reductase. Agents used for treatments are underlined and in red; the enzymes in the pathways that have been considered as targets for therapies are boxed. CAD represents three major enzymatic steps in the biosynthesis of nucleosides from glutamine, i.e., carbamoyl phosphate synthetase-II (CPS-II), aspartate transcarbamylase (ATCase) and Dihydro orotase.

Figure 1 illustrates the interconnecting networks of amino acids Pro, Gln, Asn, and Arg metabolism. We place glutamate (Glu) in the center of the networks. Glu is the product of Gln catalyzed by enzyme glutaminase (GLS) in the process known as glutaminolysis. Radiating from Glu are the connections to (i) Pro via the pyrroline-5-carboxylate (P5C) intermediate, (ii) Arg via the urea cycle, and (iii) Asn via the aspartate (Asp) intermediate catalyzed by glutamic oxaloacetic transaminase (GOT).

Figure 1 also shows that starting from Pro threading through P5C, Glu, and α-ketoglutarate (α-KG) and fumarate (in TCA cycle) reaches Arg. Then, Arg is forward-converted to ornithine (Orn) catalyzed by arginase, and then to P5C by the reversible ornithine aminotransferase (OAT). Since P5C is the precursor of Pro, this brings back to the starting Pro after a big loop. Adding to this loop is the interconnection between Glu and Asp via GOT. These metabolic wirings establish what we call the “Pro–Gln–Asn–Arg metabolic axis/loop”.

The Pro–Gln–Asn–Arg axis represents an important nodule of cancer metabolism. It occupies the major territory of amino acid metabolisms. It is also the scaffold for the biosynthesis of other non-essential amino acids and essential metabolites. Gln provides a nitrogen source of transamination involved in the production of alanine and serine, which is catalyzed by glutamic pyruvate transaminase (GPT) and phosphoserine aminotransferase 1 (PSAT1), respectively [2]. Gln is also the precursor of nucleotide biosynthesis via the CAD enzyme system (Figure 1). Glu, Asp, and Arg also directly or indirectly link to the TCA cycle that metabolizes glucose to generate ATP and reactive oxygen species (ROS) signaling. Moreover, Arg is the source of polyamine biosynthesis. These results, collectively, underscore the importance of the Pro–Gln–Asn–Arg axis/loop in cancer growth and proliferation, thus providing a molecular basis for targeted starvation therapy. Indeed, strategies of the targeted therapy of these amino acids have been in clinical development for many years. The targets (key enzymes) and agents selected for these strategies are indicated in Figure 1.

## 3. Targeting Specific Amino Acid Starvation in Cancer Therapy

### 3.1. Proline Starvation

Cellular Pro is either synthesized intracellularly or taken up by transporter-mediated degradation processes of extracellular collagen. Collagen, which consists of 25–35% of proline and 10–15% of hydroxyproline is hydrolyzed by collagenases, proteases, and peptidases. Collagen is the major component (85%) of extracellular matrix, which is an important reservoir of extracellular Pro.

Intracellular Pro is biosynthesized from two main sources: Glu and ornithine, both converge to pyrroline-5-carboxylate (P5C) intermediate. Glu converts to P5C by P5C synthetase (P5CS), and P5C reverses back to Glu by P5C dehydrogenase (P5CDH). P5C is the precursor of Pro by P5C reductases (encoded by *PYCR* genes) through the oxidation of NAD(P)H. Three *PYCR* genes encode three isozymes, among which PRCR1 and PRCY2 are mitochondrial enzymes and PRCYL is cytosolic. Proline degrades to P5C by proline dehydrogenase/proline oxidase (ProDH/Pox). ProDH/Pox is a flavin adenine dinucleotide (FAD)-containing enzyme and is tightly bound at the inner membrane of mitochondria. It functions as an electron donor through its FAD into the electron transport chain (ETC) to generate ATP and ROS [3]. Proline shuffles between mitochondria and cytosol and serves as an important redox regulator. Thus, P5C can be a precursor and product of the proline metabolism, giving rise to the so-called “proline cycle” [4].

Many studies have demonstrated the importance of Pro in cancer metabolism. Certain tumor types are intrinsically Pro-dependent for growth. The growth of these cancers can sense Pro availability. Loayza-Puch et al. [5] developed a novel approach to determine the sensing of amino acid restriction in tumor cells based on the principle of differential ribosome codon reading (diricore), deficiency of an amino acid results in tRNA deaminoacylation, and stalling of ribosome in the corresponding codon. These investigators compared diricore profiles between clear cell renal cell carcinoma (ccRCC) tissues and normal kidney tissues from the same patient and observed that tumor cells were deficient in Pro for protein synthesis [5].

Other evidence came from reports showing that overexpressed PYCR1 was associated with poor prognosis [6]. The knockout of *PYCR1* is sufficient to impair the in vivo proliferation in ccRCC cancer cells that can be negated by proline addition [5]. PCYR1 protein and mRNA levels are also elevated in human hepatocellular carcinoma (HCC) in comparison with those in the adjacent normal tissues. RNA interference of PCYR1 inhibits cell growth and promotes apoptosis accompanied with down-regulation of the c-Jun N-terminal kinase/insulin receptor substrate 1 (JNK/IRS1) pathway [7], whereas PYCRL is involved in ornithine-to-Pro biosynthesis in the cytoplasm and is linked to the pentose phosphate pathway (PPP) signaling [8].

ProDH/Pox expression is frequently reduced in tumors and may function as a survival factor for tumors under stressed challenges. However, it also functions as a tumor suppressor and is regulated by tumor suppressor p53 [3,9,10]. The ability of producing ROS may be responsible for its induction of apoptosis and autophagy function [3]. An additional study demonstrated that ProDH/Pox may promote tumor metastasis [11]. These results suggest that ProDH/Pox may regulate tumor behaviors in a cellular context-dependent manner.

P5CS encoded by the aldehyde dehydrogenase 18A1 (*ALDH18A1*) gene in the proline cycle is also frequently elevated in HCC cells, and inhibiting its expression is associated with the retardation of tumor growth [12]. Thus, it appears that PYCR1 and ProDH/Pox are attractive targets in Pro-starvation therapies of some tumor types such as ccRCC, breast cancer, and HCC. L-tetrahydro-2-furoic acid (THFA) is the first-generation inhibitor targeting the proline cycle. THFA is a proline analogue that is a known inhibitor of the ProDH/Pox enzymes. In a metastatic breast cancer animal model, treating mice bearing breast cancer tumors with THFA decreased the lung metastases with no change of primary tumor [11]. Several second-generation ProDH/Pox competitive inhibitors have recently been described, including S-5-oxo-2-tetrahydrofurancarboxylic acid (S-5-oxo) and N-propargylglycine (N-PPG), which is unique in inducing the selective mitochondrial decay of ProDH/Pox at doses that can be safely administered in vivo [13].

As for targeting PYCR1, using X ray in crystallography screening, Christensen et al. [14] recently identified five inhibitors, one of which, N-formyl-L-proline (NFLP), has a competitive (with P5C) inhibition constant of 100 µM. NFLP inhibition was shown to phenocopy the PYCR1 knockdown in breast cancer cells by inhibiting *de novo* Pro biosynthesis [15]. Pro-starvation therapy studies have gained substantial enthusiasm in recent preclinical research. No clinical Pro-starvation targeting has been conducted.

### 3.2. Glutamine Starvation

Glutamine is one of the most abundant amino acids in the body (about 500 mM, consisting about 20% of the free amino acid pool in the blood [16]). Gln is first converted to Glu and ammonium by GLS. Glu is subsequently catalyzed by glutamate dehydrogenase (GDH) and converted to α-KG. Then, α-KG enters the TCA cycle, which provides energy and macromolecular intermediates (Figure 1). Many cancer cells depend on Glu to survive, and when in scarcity, cancer cells die of apoptosis [17].

Human has two GLS, i.e., kidney-type GLS or GLS1, which has ubiquitous distribution, and liver-type GLS or GLS2, which is mainly expressed in the livers. Many reports have implicated that the elevated expression of GLS1 plays a critical role in the growth of tumors, including glioma, lymphoma, pancreatic cancer, non-small cell lung cancer, prostate cancer, and triple-negative breast cancer [18].

Glutamate can be synthesized from Pro via P5C intermediate. Moreover, Glu can be synthesized from glucose-derived α-KG and oxaloacetate (OAA) (Figure 1). This is evidenced by a study involving the knockdown of citrate synthase (CS), which is the first TCA cycle enzyme and prevents Glu-restriction-induced apoptosis because of the diversion of OAA from the TCA cycle into Asp and Asn biosynthesis. In fact, the Asn supplement can rescue Glu depletion-induced apoptosis [17].

Many human cancer cell lines are addicted to Gln and have shown sensitivity to Gln starvation, including those derived from pancreatic cancer, glioblastoma multiforme, acute myelogenous leukemia, and small cell lung cancer [19]. Given its importance in cancer metabolism, GLS has been an important target of Gln-starvation strategies. Strictly speaking, Gln starvation therapy by targeting GLS is to block Gln consumption (utilization) rather attacking Gln itself. Treating breast cancer cells with GLS inhibitors decreases downstream metabolites of Glu, including those in the TCA cycle that requires anaplerosis for replenishment [20].

Historically, Gln antagonists such as DON (6-diazo-5-oxo-l-norleucine), which inhibits several enzymes in Gln utilization, was used as an anti-tumor agent but was found to be toxic and nonspecific [21]. Later, three allosteric inhibitors of GLS have been reported: (i) BPTES ((bis-2-(5-phenylacetamido-1,2,4-thiadiazol-2-yl) ethyl sulfide 3), (ii) compound 968 (5-(3-bromo-4-(dimethylamino) phenyl)-2,2-dimethyl-2,3,5,6-tetrahydrobenzo[a]phenanthridin-4(1H)-one), and (iii) CB-839. BPTES and its analogues showed poor aqueous solubility and relatively reduced inhibitory potency [22]. Compound 968 have been shown anti-GLS activities in several cultured cell models [23]. CB-839 is an orally available, reversible noncompetitive GLS inhibitor, exhibiting anti-proliferative activity in triple-negative breast cancer cell lines and in xenografts [24]. CB-839 has been in phase I–II clinical trials against various tumor types, including, solid tumor (NCT03965845), non-small cell lung cancer (NCT04250545), and ovarian cancer (NCT03904902), either as a single agent or in combination with other anti-tumor agents (Calithera Biosciences, Inc). These studies demonstrated that CB-839 exhibits excellent tolerance, but the clinical benefits of CB-839 remain to be further investigated.

### 3.3. Asparagine Starvation

Unlike normal cells, ALL cells are unable to synthetize Asn because of asparagine synthetase (AsnS) silencing. Therefore, ALL cells become highly dependent on Asn from the circulation for survival. For the past several decades, recombinant asparaginase (ASNase) has been the mainstay for treating ALL by Asn starvation strategy.

ASNases are classified into two families: i.e., bacterial type or type I and type II, and plant-type or type III enzymes. The bacterial type II enzymes have been used in treating ALL because of their low micromolar K_m_ value and relatively easier preparation, whereas the human enzyme, a type III enzyme, has a millimolar K_m_ value for ASNase [25]. The human enzyme is poorly suitable for cancer treatment because of reduced activities. Two bacterial ASNase sources have been used in cancer treatment, i.e., those from *Escherichia coli* and *Erwinia chrysanthemi*.

Despite its effectiveness in ALL treatments, the bacterial L-ASNases have some shortcomings. (i) These enzymes have dual activities, i.e., ASNase and GLS activities. While the associated GLS activity consists only about 2–10% of the primary ASNase activity, it may enhance the overall antitumor activity, but it is thought to contribute most of the adverse toxicities in cancer treatments, including hyperglycemia, pancreatitis, and neurological seizures [26]. In a preclinical xenografts of AsnS-negative leukemia cells study, it was found that GLS activity in ASNase contributes to durable anticancer activity, whereas ASNase activity alone yields only growth delay [27]. In another study, a novel ASNase lacking the associated GLS activity showed high antitumor activities against ALL [28]. (ii) The wild-type bacterial enzymes are subjected to degradation in the circulation. To increase the stability of the enzyme, Pegylated ASNase has been formulated (Oncasper, an *E. coli* ASNase) to boost the half-life in blood, presumably by protecting proteolytic attack of the bioconjugate [29]. However, increasing the half-life of ASNase in blood may also sustain ammonia levels that may be toxic.

Following Asn starvation, AsnS is frequently activated, resulting in the development of resistance to ASNase [5]. AsnS catalyzes the conversion of Asp and Gln to Asn and Glu in an ATP-dependent reaction [30]. The activation of AsnS follows the mechanism of general amino acid response. This response actives elf2 kinase. The phosphorylation of elf2 by the kinase general control nonderepressible (GCN2) suppresses global protein synthesis, but intriguingly, it increases the translation of a small subset of mRNA, including the activating transcription factor 4 (ATF4). ATF4 binds to an enhancer at the *AsnS* promoter and induces its expression. Alternatively, elf2 phosphorylation can be activated via endoplasmic reticulum stress-induced unfolded protein response (UPR) via PKR-like endoplasmic reticulum kinase (PERK), resulting in the transcriptional activation of *AsnS* [30]. Primary childhood ALL patients express AsnS very little or not at all because of epigenetic control by DNA methylation at the CpG islands in the promoter of *Asn*S. This prevents the accessibility of ATF4 to turn on its expression [31,32], thereby maintaining reduced levels of Asn and sensitivity to ASNase treatments.

An additional important pathway that collaborates with the GCN2-p-elf2 pathway in regulating ATF4 in response to nutrient stresses is the Kras–PI3K–AKT–NRF2 (nuclear factor erythroid 2) signal pathway [33]. Kras regulates cell growth in response to Asn and Glu availabilities. PI3K up-regulates NRF2 (nuclear factor erythroid 2) to support amino acid homeostasis through ATF4. It regulates the binding of ATF4 to the promoter of *AsnS* in the absence of its active inhibitor Keap1 [34]. NRF2 is generally a stress-responsive transcription factor that regulates a wide variety of genes involved in anabolic cancer metabolism, especially glucose and Gln metabolism.

The findings that low AsnS expression sensitized to ASNase in ALL patients prompted investigations on other cancer types, but the results are complex [35]. The expression of AsnS in tumor generally is invertedly correlated with ASNase sensitivities even in solid cancers such as HCC [36] and pancreatic cancer cells [37]. However, it was reported that breast cancer cells with high AsnS levels have greater potential to metastasize to the lung, which is accompanied with an upregulation of genes involved in epithelial-to-mesenchymal transition (EMT) function [38]. Overall, ASNase has shown clinical activity in ALL and some lymphomas but not others. The underlying reasons for not being effective in other tumors are not completely understood, perhaps because of the little or infrequent up-regulation of AsnS in ALL-patients treated with ASNase [39,40], and the severe adverse effects outweigh the benefits in solid cancers treatments [41].

### 3.4. Arginine Starvation

Cellular Arg can be obtained from an extracellular source carried out by several cationic amino acid transporters (CATs), especially CAT-1 and CAT2B, which have higher Arg binding affinities than others [42], and intracellularly from proteasomal and lysosomal protein breakdown. However, the majority of Arg is derived from *de novo* biosynthesis catalyzed by two sequential enzymes: argininosuccinate synthetase (ASS), which condenses citrulline and Asp into argininosuccinate (AS), followed by arginosuccinate lyase (ASL), which converts AS into Arg and fumarate (Figure 1). ASS and ASL are the rate-limiting enzymes, and citrulline is the limiting substrate for *de novo* Arg biosynthesis (Figure 1). Arg is a remarkable amino acid in that its deficiency inside the cells can be sensed by an increasing up-regulation of ASS1 expression; and the synthesis of Arg is shut down when cellular Arg levels are elevated by the suppression of ASS1 expression. Many human malignancies do not produce a sufficient amount of Arg because of ASS1 silencing. These tumors normally acquire extracellular Arg for survival, providing a metabolic vulnerability for targeted Arg-starvation therapy using Arg-depleting recombinant proteins such as pegylated arginine deiminase (ADI-PEG20) and human arginase 1 (Figure 1). ASS1 digests Arg into citrulline and ammonia. Arginase 1 catalyzes the conversion of Arg into urea and ornithine, which is the precursor of P5C (Figure 1). In terms of Arg deprivation efficiency, the human arginase is only a fraction (1/1000 times) of the bacterial enzyme [43].

Many early reports showed that ASS1-silencing in tumors is epigenetically controlled by DNA methylation at the *ASS1* promoter [44,45,46] and a DNA-demethylation agent such as 5′ aza-2′-deoxycystine (Aza-dC) can induce ASS1 expression [45,47]. In a clinical setting, Nicholson et al. [48] reported that aberrant methylation in the *ASS1* promoter correlated with the transcriptional silencing of ASS1 in ovarian cancer cells. Epigenetic DNA methylation in ASS1 silencing was also reported in nasopharyngeal carcinoma [49], malignant mesothelioma [50], glioblastoma [45], bladder cancers [51], myxofibrosarcomas [47], and lymphoma [44]. However, other reports showed that the extents of methylated CpG islands at the *ASS1* and *ASL* promoters were not always correlated with ASS1 mRNA expression levels [52].

**Figure 2:**
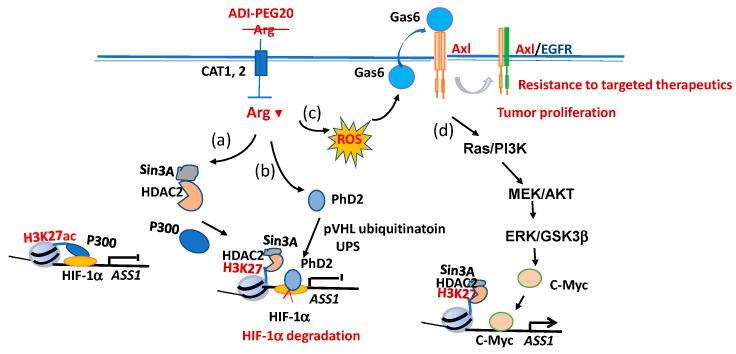
Mechanisms of argininosuccinate synthetase (ASS1) induction by Arg starvation. Arg-depleting recombinant proteins such as pegylated arginine deiminase (ADI-PEG20) digests extracellular Arg, resulting in the depletion of intracellular Arg. (**a**) Before ADI-PEG20 treatment, ASS1 is silenced by HIF-1α binding to its promoter due to the association of histone acetyltransferase p300, which acetylates H3K14ac and H3K27ac (for simplicity, only H3K27ac is indicated). ADI-PEG20 treatment prompts P300 dissociation from the promoter, allowing histone deacetylase HDAC2 and co-factor Sin3A to deaceacetylate histone H3K27ac and H3K14ac. (**b**) Propyl hydroxylase (PhD2)–pVHL ubiquitine–proteosomal system moves in to the *ASS1* promoter and destroys HIF-1α (see reference 54). (**c**) In the meantime, Arg deprivation generates reactive oxygen species (ROS), which triggers gas6 externalization to interact with the Axl receptor. (**d**) Axl activates the signal transduction involving Ras/PI3K, MEK/AKT and ERK/GSK3b resulting in the stabilization of c-Myc, which is a positive transcription factor to turn one *ASS1* expression [55]. Then, elevated ASS1 feedbacks to suppress c-Myc and Axl signaling.

Our group reported that ASS1 silencing is transcriptionally controlled by the negative regulator HIF-1α, which binds the E-box (5′-CACGTA) located at the promoter of the *ASS1* gene [53,54]. Promoter occupancy is associated with histone acetylation at H3K14 and H3K27 by the histone acetyltransferase (HAT) p300. Within minutes after cells are exposed to Arg-depleting conditions, p300 falls off from the promoter. In the meantime, histone deacetylase HDAC2 and its co-factor Sin3A move in and deacetylate H3K14ac and H3K27ac. This chromatin remodeling allows the propyl hydroxylase (PHD2)–pVHL–lead ubiquitin–proteasomal complex to approach the *ASS1* promoter and degrades HIF-1α in loco, thereby providing accessibility of the positive regulator c-Myc to interact with the E-box and turn on ASS1 expression [54]. These results demonstrated that ASS1 silencing and subsequent de-silencing are controlled by epigenetic mechanisms (Figure 2a,b).

Positive transcriptional and pos-translational mechanisms are involved in ASS1 up-regulation under Arg-depleting conditions. This was first initiated by the externalization of Gas6, which is the ligand of receptor tyrosine kinase (RTK) Axl (Figure 2c). In the untreated cells, Gas6 is associated with the inner membrane of the plasma membrane. Externalized Gas6 binds to the extracellularly localized ligand-binding domain of Axl and activates the downstream intracellular Ras/PI3K/Akt growth signal, leading to the accumulation of c-Myc, first by protein stabilization [55,56]. Since c-Myc is known to transcriptionally self-regulate [57], this results in amplifying the c-Myc level. Similar to HIF-1α, c-Myc is also an E-box binder, but c-Myc is a positive regulator and turns on the expression of ASS1. The elevated expression of ASS1 feedbacks to suppress c-Myc and Axl expression, thereby shutting down ASS1 expression and abolishing Arg starvation [55]. In addition to Axl, c-Myc also regulates other RTK such as EphA2. Collaboration between c-Myc and EphA2 results in conferring ADI-resistant cells to cross-resistance with EGFR inhibitors [58]. Recently, we found that Aza-dC induced ASS1 expression by the down-regulation of HIF-1α but up-regulated c-Myc. We further demonstrated that the clock protein DEC1 is the master regulator of HIF-1α and c-Myc, which regulate ASS1 [59]. These data offer an alternative explanation on how the DNA de-methylating agent induces ASS1 expression. Taken together, Arg starvation regulation of ASS1 expression involves complexed epigenetic and genetic, transcriptional and post-transcriptional, intracellular and extracellular signaling.

ADI-PEG20 has been used in many clinical investigations against a variety of tumor types (for reviews, see ref [60,61]). A recent phase III study involved 635 HCC patients treated with ADI-PEG20 monotherapy, and the results show that while the treatment was safe, it did not exhibit any significant overall survival benefit for HCC [62]. These results suggest that the further development of ADI-PEG20 is required.

Pegylated recombinant human arginase (Peg-rhArg1) has been in clinical trials on HCC [63]. While the overall survival rates remained low, it was found that progression-free survival (PFS) was significantly longer for a subset of patients with adequate Arg depletion. In another study, it was found that human acute myeloid leukemia (AML) is highly dependent on Arg for survival, and therefore is Arg auxotrophic because of reduced expression of ASS1. AML is very sensitive to the treatment with pegylated Arg1 (BCT-100) [64]. Nevertheless, similar to ADI-PEG20, further development of recombinant Arginase1 in clinical use is needed.

## 4. Common Mechanisms Associated with Pro- Gln- Asn- and Arg-Starvation Therapies

### 4.1. Production of ROS

Reactive oxygen species are highly reactive molecules that are produced as by-products of metabolism, especially when cancer cells are under nutritional stresses. Deprivations of any one of Pro, Gln, Asn, or Arg amino acids all produce ROS, mostly of mitochondrial origin.

ROS can be beneficial or detrimental to cells, depending on the levels and locations of the production and cellular context. At low levels, ROS facilitates cancer cell survival by an enhanced activation of growth factors and RTK [65]. One example is our finding found that within minutes of Arg depletion with ADI-PEG20, ROS production can be detected. The produced ROS drives the Gas6-Axl growth signal, which is initiated by inducing Gas6 externalization, perhaps by changing Gas6 conformation through alternations of intracellular cysteine bisulfide linkages [55]. This signal leads to the transcriptional activation of ASS1 that elicits ADI-PEG20 resistance, as described above [66,67,68].

On the other hand, high levels of ROS are detrimental by suppressing tumor survival through the induction of apoptosis. High Pro levels protect cancer cells from oxidative stress during oncogenesis. Pro has antioxidant activity that suppresses the production of ROS [15,69,70]. It has been reported that overexpressing ProDH/Pox reduces cellular Pro levels but increased ROS. In contrast, overexpressing P5CS, which increases Pro, is associated with reduced ROS and cell survival [15].

ProDH/Pox is an inner mitochondrial membrane flavin-dependent enzyme that donates electrons through flavin adenine dinucleotide (FAD) to the electron transport chain for ATP generation. As a result of its shared localization with the mitochondrial respiratory electron transfer chain (ETC), which comprises Complex I to IV, ProDH/Pox interacts with the ETC complexes [71]. Under stressed conditions, electron transfers may directly target oxygen and form superoxide radicals and other ROS. ProDH/Pox induces programmed cell death by stimulating the expression of tumor necrosis factor-related apoptosis activated ligand (TRAIL), DR5, and cleavage of caspase-8, and it also activates caspase-9 and caspase-3 [72].

Glutamine is the precursor of glutamate, and it converts by GDH to α-KG, which is an important intermediate in the TCA cycle in the mitochondrion, the power house of ATP production and ROS generation under metabolic stress. The concept of “Glutamine addiction” in cancer cells also underscores the importance of ROS production under Gln starvation stress [66]. Pancreatic cancer cells treated with GLS inhibitor CB-839 induce a robust oxidative stress with increases of ROS at an early time point of treatment [73]. Moreover, Gln is the precursor of Glu, which is the substrate of glutathione (GSH) biosynthesis. GSH is the most abundant physiological antioxidant in all eukaryotic cells. Therefore, Gln deprivation may cause increased ROS due to GSH suppression [66,74]. Indeed, reducing cellular Gln levels during Gln withdrawal [75], GLS knockdown, or exposure of cells to the GLS inhibitor CB-839 all resulted in a robust induction of ROS in high GLS-expressing but not in low GLS-expressing ovarian cancer cells [76], further supporting the roles of GLS in ROS production

The involvement of ROS as signal transduction molecules in nutrient starvation-induced autophagy has long been elucidated [77,78,79]. Autophagy is a process that eliminates bulk intracellular damaged constituents for recycling into cellular building blocks. This process is crucial for cell survival. However, autophagy can also induce cell death through catastrophic damages of cellular constituents. Chen et al. [80] reported that treating glioblastoma cells with ASNase induces ROS production and activates autophagy, which was significantly reduced by antioxidant NAC. One important target in the ROS-regulated autophagy is Atg4, which contains conserved cysteine residues at Cys^77^ and Cys^81^. As the autophagosome matures toward fusion with lysosome, redox regulated Atg4 promotes the lipidation of Atg8, which is an essential step in the process of autophagy [81]. The suppression of autophagy with inhibitor chloroquine (CQ) and LY294002 enhanced ASNase-killing cells via caspase-dependent apoptosis. Similar results were described for laryngeal squamous cell carcinoma cells [82]. Arg depletion also induces “atypical autophagy” in prostate cells as reported by Changou et al. [83]. These results suggest that amino acid deprivation can induce apoptosis and autophagy.

Cutaneous melanoma has high incidences of BRAF mutation at V600E (40–60%); thus, BRAF inhibitors vemurafenib and dabrafenib are effective in cutaneous melanoma treatments. However, resistance almost inevitably occurs [84,85]. BRAFi-resistant cells still maintain their sensitivity to ADI-PEG20 treatment because of low ASS1 expression. Intriguingly, we found that BRAFi-resistant cells exhibit defects in response to Arg-starvation-induced autophagy because of the suppression of AMPK-α1 [86,87]. AMPK activation promotes autophagy by means of activation of ULK complex at serine 317 (S317) and serine 777 (S777), which in turn activates beclin for triggering autophagosome formation [78].

### 4.2. Involvements of the Same Transcription Regulators

Nutritional stresses under Pro, Gln, Asn, and Arg deprivations alter global gene expression profiles. The proteomic profiling of Arg-deprivation response revealed thousands of genes involved in metabolic reprograming between drug-resistant variants and their parental counterpart [88]. Metabolomic profiling of bone marrow and peripheral blood specimens obtained from ALL patients before and after Peg-ASNase treatment revealed global changes of metabolites [89]. Metabolic systems of colon and lung cells treated with CB-839 also revealed substantial differences in intracellular metabolites [90]. Likewise, RNA sequencing and secretory proteins analyses revealed altered genes expression associated with Pro starvation [91]. These findings are not surprising, but do they involve common transcriptional regulators? Here, we discuss two transcription factors that are involved in regulation of important genes in the Pro–Gln–Asn–Arg axis/loop.

#### 4.2.1. c-Myc

C-Myc is an important transcriptional factor. It regulates an estimate of about 15% of overall human genes [92]. In ChIP-Seq analyses, approximate 7000 binding sites in Burkitt lymphoma cells were estimated [77]. Myc activation/amplification is frequently associated with progression of a wide variety of human cancers [68].

**Figure 3:**
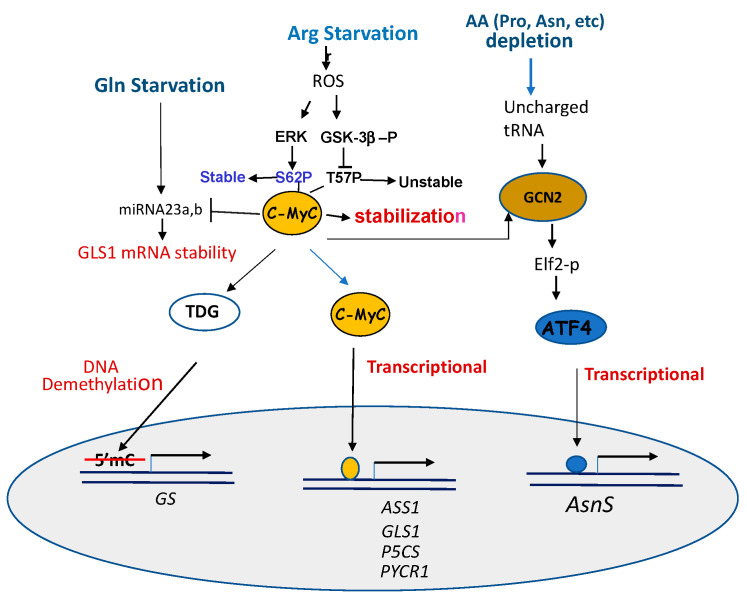
Multiple mechanisms of c-Myc upregulation and transcriptional induction of target genes under different amino acid-starvation conditions. Under Gln starvation, c-Myc downregulates miRNA 23a,b resulting in the stabilization of target GLS1 mRNA. Under Arg starvation, the induced ROS promotes the phosphorylation of c-Myc at S62P via an ERK intermediate and an inhibition of T57P phosphorylation, resulting in c-Myc stabilization. C-Myc also cross-talks with GCN2 in the amino acid depletion signaling, resulting in increased translation of ATF4. Both ATF4 and c-MyC can directly bind to the promoter of their targeting genes. Alternatively, c-Myc can up-regulate thymine DNA glycosylase (TDG) to induce DNA demethylation at the promoter of GS.

Mechanisms of c-Myc activation in responding to amino acid starvation are multiple (Figure 3).

First, c-Myc is known to control the expression of many amino acid transporters [93]. For example, c-Myc transcriptionally regulates *ASCT2*, *SN2* [68], and *SLC1A5* [94] Gln transporters by directly binding to their promoters [68]. Second, c-Myc transcriptionally regulates genes encoding enzymes involved in the syntheses of important metabolites in the Pro–Gln–Asn–Arg network by directly binding to their promoters. These include genes encoding GLS [67,68], P5CS and PYCR1 [67], and ASS1 [55,95]. However, c-Myc down-regulates the expression of ProDH/POX, P5CDH, and GS in the pathway from Pro to Glu [96]. Third, c-Myc regulates the major amino acid stress response pathway via the GCN2–ATF4 axis [97]. Fourth, c-Myc post-transcriptionally regulates genes involving DNA demethylation, resulting in an activation of the gene. While many breast cancer cell lines express high levels of GS and c-Myc, however, in one breast cell line expressing reduced levels of GS, it was found that Gln restriction activates c-Myc transcriptionally targeting thymine DNA glycosylase (TDG), which induces demethylation [98] of the *GS* promoter, resulting in an induction of GS express, just like treating these cells with DNA methyltransferase inhibitor Aza-dC [99].

Fifth, Gao et al. reported the link between c-Myc and Gln metabolism. Elevated c-Myc protein transcriptionally suppresses two miRNA23a,b, which target the GLS mRNA 3′ UTR sequences for degradation. The inhibition of these miRNAs by c-Myc results in an up-regulation of mitochondrial GLS1, which in turn increases the glutaminolysis and production of ROS [100]. The regulation of GLS by c-Myc via miRNA23a,b is also reported in another study [101]. This is an example in which c-Myc regulates GLS1 via a post-transcriptional mechanism. One the other hand, c-Myc suppresses the expression of ProDH/Pox by up-regulating miRNA23b*, which shares the same transcription origin of miRNA23b. C-Myc was found to up-regulate Agonaute 2 protein (Ago 2), which is a key player of miRNA-regulated mRNA stability [102].

c-Myc protein is normally targeted for ubiquitin proteasomal degradation by phosphorylation at serine 62 (S62) and threonine 58 (T58). S62 is the target of ERK and T58 is the target of GSK-3b. We found that in ADI-PEG20-treated cells, the ERK-mediated phosphorylation of S62 prevents c-Myc from degradation, whereas GSK-3b phosphorylated T-58 promotes c-Myc degradation. GSK-3b itself is the target of PI3K/AKT-mediated phosphorylation at Ser9. GSK-3b phosphorylation inactivates its ability of phosphorylating c-Myc (T58). These result in the stabilization of c-Myc. Thus, ADI-PEG20 treatment enhances post-translational modifications of c-Myc stability through the ERK and PI3K/AKT/GSK-3b signals [56] (Figure 3).

#### 4.2.2. ATF4

Activating transcription factor 4 (ATF4) is a stress-induced transcription factor regulating a wide variety of genes involved in cellular adaptation to unfavorable growth conditions, such as ER stress resulting from unfold protein response (UPR) and the amino acid response (AAR). ATF4 is activated in response to each of Pro [103], Gln [23,33], Asn [73] and Arg starvations [104], following the conserved AAR signal of GCN2-p-eIF2-ATF4. However, ATF4 is also a target of c-Myc via GCN2 kinase [97] (Figure 3), indicating the cross-talk between two important transcriptional regulation pathways in general amino acid starvation response.

ATF4 is a basic leucine-zipper family protein. It forms a homodimer or heterodimers with other family members. Through the combination of heterodimerization with other members in the family, ATF4 transcriptionally regulates a large scope of target genes that are involved in amino acid transport, metabolic adaption, cell cycle arrest, apoptosis, and autophagy [105]. Similar to c-Myc, ATF itself is subjected to post-translational modification, such as polyubiquitination that affects its stability under nutritional (Gln) stress [106]. In AAR, ATF recruits the p300/CBP-associated factor and transcriptionally activates the expression of its target genes [107]. AsnS silencing in ALL cells is mainly due to DNA hypermethylation at several CpG islands, preventing the promoter binding of ATF4. However, as mentioned above, Asn starvation by ASNase induces the demethylation of these CpG islands and allows promoter binding of ATF4 to turn on the *AsnS* gene [31]. These results suggest that the master regulator ATF4 controls multiple layers of gene regulation in response to amino acid starvations.

## 5. Mechanisms of Drug Resistance

### 5.1. Reprogramming of Survival Amino Acid Metabolism

Cancer cells are notoriously capable for exploiting survival mechanisms under metabolic stresses. This may be particularly relevant when the pathways are intermingled in complex networking. Cancer cells often evolve to use alternative strategies to overcome drug resistance.

#### 5.1.1. Re-Activation of the Silenced Genes

Re-activating the silenced gene involved in amino acid auxotroph is the most straightforward mechanism of treatment resistance associated with amino acid-starvation therapies. As alluded above, the reactivation of ASS1 expression is a common mechanism of ADI-PEG20 resistance [88,108]. Leukemic cells sensitive to ASNase are most often due to a reduced expression of AsnS. One important mechanism of ASNase resistance is associated with an increased expression of AsnS. Targeting Gln starvation using the GLS1 inhibitor BPTES resulted in the elevated expression of GLS1 [109]. Likewise, Pro depletion is associated with the up-regulation of PYCR1, which is the key enzyme for the biosynthesis of Pro [5,14]. The re-expression of these once-silenced genes is to replenish the needed amino acids that cause the starvation.

#### 5.1.2. Compensatory Activation and Cross-Interference of Metabolic Pathways

We previously investigated the metabolic reprogram in ADI-PEG20-resistant melanoma cell lines, which was selected with prolonged exposure to the drug. We observed that these resistant variants display enhanced glucose transporter 1 and lactate dehydrogenase-A expression but reduced the expression of pyruvate dehydrogenase and elevated sensitivity to the glycolytic inhibitors. Furthermore, the resistant cells showed elevated GDH and GLS expression and were preferentially vulnerable to Gln inhibitors [95]. These results suggest a metabolic reprogramming toward Gln addiction and glucose dependence during the development of drug resistance. Results of recent proteomic analyses demonstrated that the activation of multiple metabolic processes is an adaptive measure for cells to survive under Arg starvation [88].

It was found that in the kidney cancer cells, depleting Asn by ASNase activates the expression of PYCR1 for tumor growth [5], which is a key enzyme in Pro production. This finding suggests a compensatory mechanism of metabolic adaptation. Indeed, PYCR1 is induced by Glu shortage, suggesting that the compensatory pathway may follow the Asn–Asp–Glu–P5C pathway to activate PYCR1 to promote the biosynthesis of Pro (see Figure 1).

In the pancreatic cancer cells treated with GLS inhibitor CB-839, using uniformly ^13^C-labeled Gln to trace the itinerary of Gln-derived carbons in control versus CB-839-treated cells, it was discovered that it increased unlabeled Glu in the treated cells, indicating alternative pathways for Glu supply [73]. While the alternative pathways were not reported in this study, other work identified that during Gln limitation, Asn could support the adaptation of tumors to Gln limitation by the induction of GS [110]. Another alternative pathway may be the p53-regulated Arg transporter Slc7a3, which enables cancer cells to adapt Gln deprivation [111]. Collectively, these results suggest the compensatory support of metabolic pathways under amino acid deprivation conditions. These results may also suggest that combined targeting Gln and Asn using GB-839 and ASNase strategies may overcome resistance that would otherwise develop to either one alone. Of course, the increased cytotoxicity in combination treatment also needs to be taken into consideration.

In addition to the compensatory effect, recent studies have demonstrated cross-talks between Arg and Gln starvation signaling. Our laboratories reported that the knockdown of ASS1 resulted in increased sensitivities to both Arg- and Gln-starvation stresses, whereas increased ASS1 expression by ectopic transfection was associated with resistance to both Arg and Gln starvation. Moreover, supplementing with permeable fumarate, a metabolite downstream of α-KG in the TCA cycle resulted in the down-regulation of ASS1 expression and increased sensitivity to both Arg- and Gln-deprivation treatments [108]. The shared sensitivity/resistance between Arg- and Gln-starvation is a vivid example of the multiple effects that even include targeting an individual amino acid starvation.

### 5.2. Roles of Amino Acid Transporters

It is conceivable that amino acid transporters play important roles in regulating drug sensitivity/resistance in amino acid starvation therapies; after all, they control the inputs of cellular amino acid contents for cell growth. Here are just a few examples: SNAT1 (SLC38A1), SNAT2 (SLC38A2), and ASCT2 (SLC1A5) are the major Gln transporters in cancer cells [112]. SNAT2 senses the availability of substrate and increases its expression when cellular amino acid substrates are low [113]. Gln restriction induces the expression of SNAT2 transporter [114]. The expression of SNAT1 is greatly enhanced when another transporter ASCT2 is silenced [115]. These results suggest compensatory interactions between amino acid transporters.

The CAT family (SLC7A1,2,3) consists of a cation amino acid transporter that transports positively charged amino acids such as Arg and Lys. CAT1 expression is induced by AAR via ATF4 [116]. In another study, it was reported that Gln starvation activates p53, which induces the transcriptional up-regulation of the CAT3 (*SLC7A3)*, and its expression promotes an increase of intracellular Arg levels for cell growth [111]. The uptake of Arg by these transporters largely depends on the intracellular Arg contents and amino acid compositions [42].

It is important to note that some amino acid transporters function as exchangers and in which case, cellular levels of one amino acid can regulate levels of other amino acids. Asn levels regulate the transport of other amino acids such as Arg, serine, and histidine in supporting mTORC1 activity for protein synthesis [117]. Likewise, Gln sensitivity is also regulated by a bidirectional amino acid transporter SLC7A5/SLC3A2, which regulates the simultaneous efflux of L-Glu out of cells and the transport of other amino acids into cells [118].

### 5.3. Induction of Immunogenic Reactions Associated with Using Microbial Enzymes

#### 5.3.1. ADI-PEG20

ADI-PEG20 was derived from microorganisms; therefore, it is unstable in the circulation and immunogenic. Its half-life is increased after pegylation, but its antigenicity remains. Immunogenicity is an intrinsic problem associated with the use of foreign recombinant protein. In a phase I clinical trial against patients with high-grade glioma, using combination therapy with cisplatin and pemetrexed, it was found the serum Arg levels were rapidly decreased followed by increased anti-ASS1 antibody [119]. Similar kinetics were also observed in another trial of HCC using ADI-PEG20 monotherapy [120]. High levels of anti-ADI antibody persisted for several weeks. It may neutralize ADI activities and contribute to the treatment failure.

#### 5.3.2. Recombinant Asparaginases

Two ASNase sources have been used in treating ALL, i.e., native enzymes derived from *Escherichia coli* and *Erwinia chrysanthemi*, and pegylated *E coli* ASNase. The development of anti-ASNase antibodies is mostly observed with native *E. coli* ASNase, generally ranging 10–30% but can be up to 75% of ALL patients, depending upon ASNase preparations and treatment [121]. Patients who encountered hypersensitivity to one preparation may cause the rapid inactivation of ASNase, leading to worse prognosis or so-called “silent inactivation” [122]. The availability of multiple ASNase preparations allows patients to switch to alternative ASNases. In a study involving 1155 high-risk ALL children treated with pegylated *E. coli* ASNase, which replaced native ASNase post-induction treatment, the 5-year event-free rates were not different between patients with a negative versus positive antibody titer [123]. However, the overall survival rates of ALL have been about 85%, which were achieved mainly due to the use of intensive and prolonged ASNase therapy [124,125]. Thus, it appears that toxicities associated with ASNase in ALL patients are manageable.

## 6. Conclusions and Perspective

The identifications that malignant human cells require specific amino acids from extracellular milieu to support their proliferation needs provide the metabolic basis of targeting amino acid starvation therapy. The success of ASNase in Asn-starvation therapy of treating ALL [126] has motivated the development of targeted amino acid restrictions in other cancer therapies. Extensive clinical efforts have been conducted in Arg- and Gln-starvation trials, whereas targeting Pro-starvation remains at preclinical development. Amino acid starvation therapies remain promising for the reasons that we have learned a great deal along the way. However, challenges remain. Chiefly, clinical resistance to the treatments remains the bottleneck that needs to be overcome. First, *E. coli*-derived ASNase in Asn-starvation and *Mycoplasma*-derived ADI-PEG20 in Arg-starvation therapy are of microorganism origins that have an inherent immunogenicity issue. Moreover, treatments with ASNase and ADI-PEG20 induce AsnS and ASS1 re-expression, respectively, rendering treatment failure. The efficacies of these treatments may be improved as we know more about the underlying mechanisms of AsnS and ASS1 induction. The signal mechanisms underlying how ADI-PEG20 induces ASS1 expression have been extensively characterized (Figure 2). We found that RTK, PI3K/AKT, and P300/HDAC are important components in the pathways associated with ADI-PEG20-induced ASS1 expression. Many clinically approved inhibitors targeting these components are available that can be used in combination with ADI-PEG20 for cancer therapy.

Clinical studies of Gln-starvation therapy have been largely focused on the use of a GLS inhibitor such as CB-839, which has been through phase I/II clinical evaluations. While the final outcome of CB-839 remains to be investigated, new and potent GLS inhibitors have been synthesized based on structural characterizations and molecular modeling [127,128]. These small anti-GLS molecules may have great potential as antitumor agents. However, thus far, this line of research has concentrated on targeting GLS; future studies may need to explore new targets for opening up the opportunities.

Many lines of evidence suggest that pro availability affects cancer growth and proliferation, supporting the strategies of targeting Pro-starvation development [129]. Two enzymes are particularly relevant: PYCR1 has been shown to play a positive role in supporting cancer cell survival [12,130], whereas ProDH/POX has been shown displaying pro-tumor or anti-tumor properties, depending upon the contexts of microenvironment and cancer type [4]. These two enzymes have been considered as potential targets for clinical applications. While several Pro analogues have been shown activities against PYCR1 in cultured cell models, no clinical evaluation of these inhibitors has been conducted. Nevertheless, given time, it is anticipated that effective Pro-starvation strategies may come along.

Finally, one important issue associated with targeted specific amino acid starvation cancer therapy is the activation of compensatory pathways that allow cancer cells to reprogram their survival advantages. We have devoted a substantial portion in this review to address the interconnecting networking involved in the Pro–Gln–Asn–Arg metabolic axis/loop. This effort has provided a broader scope of targeting amino acid starvation beyond the currently individual one. Perhaps it is time to think globally when designing strategies to targeted amino acids starvation therapy. This may eventually lead to the development of effective strategies in cancer treatment.

## References

[1] Yahyaoui R., Pérez-Frías J. (2019). Amino Acid Transport Defects in Human Inherited Metabolic Disorders. Int. J. Mol. Sci..

[2] Coloff J.L., Murphy J.P., Braun C.R., Harris I.S., Shelton L.M., Kami K., Gygi S.P., Selfors L.M., Brugge J.S. (2016). Differential Glutamate metabolism in proliferating and quiescent mammary epithelial cells. Cell Metab..

[3] Liu W., Phang J.M. (2012). Proline dehydrogenase (oxidase) in cancer. BioFactors.

[4] Tanner J.J., Fendt S.-M., Becker D.F. (2018). The Proline Cycle as a Potential Cancer Therapy Target. Biochemistry.

[5] Loayza-Puch F., Rooijers K., Buil L.C.M., Zijlstra J., Vrielink J.F.O., Lopes R., Ugalde A.P., Van Breugel P., Hofland I., Wesseling J. (2016). Tumour-specific proline vulnerability uncovered by differential ribosome codon reading. Nat. Cell Biol..

[6] Gao Y., Luo L., Xie Y., Zhao Y., Yao J., Liu X.-L. (2020). PYCR1 knockdown inhibits the proliferation, migration, and invasion by affecting JAK/STAT signaling pathway in lung adenocarcinoma. Mol. Carcinog..

[7] Zhuang J., Song Y., Ye Y., He S., Ma X., Zhang M., Ni J., Wang J., Xia W. (2019). PYCR1 interference inhibits cell growth and survival via c-Jun N-terminal kinase/insulin receptor substrate 1 (JNK/IRS1) pathway in hepatocellular cancer. J. Transl. Med..

[8] Phang J.M., Liu W., Hancock C.N., Fischer J.W. (2015). Proline metabolism and cancer. Curr. Opin. Clin. Nutr. Metab. Care.

[9] Polyak K., Xia Y., Zweier J.L., Kinzler K.W., Vogelstein B. (1997). A model for p53-induced apoptosis. Nat. Cell Biol..

[10] Liu W., Glunde K., Bhujwalla Z.M., Raman V., Sharma A., Phang J.M. (2012). Proline Oxidase Promotes Tumor Cell Survival in Hypoxic Tumor Microenvironments. Cancer Res..

[11] Elia I., Broekaert D., Christen S., Boon R., Radaelli E., Orth M.F., Verfaillie C., Grünewald T.G.P., Fendt S.-M. (2017). Proline metabolism supports metastasis formation and could be inhibited to selectively target metastasizing cancer cells. Nat. Commun..

[12] Ding Z., Ericksen R.E., Escande-Beillard N., Lee Q.Y., Loh A., Haines R.L., Steckel M., Haegebarth A., Ho T.S.W., Chow P.K.H. (2020). Metabolic pathway analyses identify proline biosynthesis pathway as a promoter of liver tumorigenesis. J. Hepatol..

[13] Scott G.K., Yau C., Becker B.C., Khateeb S., Mahoney S., Jensen M.B., Hann B.C., Cowen B.J., Pegan S.D., Benz C.C. (2019). Targeting Mitochondrial Proline Dehydrogenase with a Suicide Inhibitor to Exploit Synthetic Lethal Interactions with p53 Upregulation and Glutaminase Inhibition. Mol. Cancer Ther..

[14] Christensen E.M., Bogner A.N., Vandekeere A., Tam G.S., Patel S.M., Becker D.F., Fendt S.-M., Tanner J.J. (2020). In crystallo screening for proline analog inhibitors of the proline cycle enzyme PYCR. J. Biol. Chem..

[15] Krishnan N., Dickman M.B., Becker D.F. (2008). Proline modulates the intracellular redox environment and protects mammalian cells against oxidative stress. Free Radic. Biol. Med..

[16] Altman B.J., Stine Z.E., Dang C.V. (2016). From Krebs to clinic: Glutamine metabolism to cancer therapy. Nat. Rev. Cancer.

[17] Zhang J., Fan J., Venneti S., Cross J.R., Takagi T., Bhinder B., Djaballah H., Kanai M., Cheng E.H., Judkins A.R. (2014). Asparagine Plays a Critical Role in Regulating Cellular Adaptation to Glutamine Depletion. Mol. Cell.

[18] Shah R., Chen S. (2020). Metabolic Signaling Cascades Prompted by Glutaminolysis in Cancer. Cancers.

[19] Wise D.R., Thompson C.B. (2010). Glutamine addiction: A new therapeutic target in cancer. Trends Biochem. Sci..

[20] Singleton D.C., Dechaume A.-L., Murray P.M., Katt W.P., Baguley B.C., Leung E. (2020). Pyruvate anaplerosis is a mechanism of resistance to pharmacological glutaminase inhibition in triple-receptor negative breast cancer. BMC Cancer.

[21] Lemberg K.M., Vornov J.J., Rais R., Slusher B.S. (2018). We’re Not “DON” Yet: Optimal Dosing and Prodrug Delivery of 6-Diazo-5-oxo-L-norleucine. Mol. Cancer Ther..

[22] Shukla K., Ferraris D.V., Thomas A.G., Stathis M., Duvall B., Delahanty G., Alt J., Rais R., Rojas C., Gao P. (2012). Design, Synthesis, and Pharmacological Evaluation of Bis-2-(5-phenylacetamido-1,2,4-thiadiazol-2-yl)ethyl Sulfide 3 (BPTES) Analogs as Glutaminase Inhibitors. J. Med. Chem..

[23] Lampa M., Arlt H., Christopher W., Ospina B., Reeves J., Zhang B., Murtie J., Deng G., Barberis C., Hoffmann D. (2017). Glutaminase is essential for the growth of triple-negative breast cancer cells with a deregulated glutamine metabolism pathway and its suppression synergizes with mTOR inhibition. PLoS ONE.

[24] Gross M.I., Demo S.D., Dennison J.B., Chen L., Chernov-Rogan T., Goyal B., Janes J.R., Laidig G.J., Lewis E.R., Li J. (2014). Antitumor Activity of the Glutaminase Inhibitor CB-839 in Triple-Negative Breast Cancer. Mol. Cancer Ther..

[25] Nomme J., Su Y., Lavie A. (2014). Elucidation of the Specific Function of the Conserved Threonine Triad Responsible for Human l-Asparaginase Autocleavage and Substrate Hydrolysis. J. Mol. Biol..

[26] Baruchel A., Brown P., Rizzari C., Silverman L., Van Der Sluis I., Wolthers B.O., Schmiegelow K. (2020). Increasing completion of asparaginase treatment in childhood acute lymphoblastic leukaemia (ALL): Summary of an expert panel discussion. ESMO Open.

[27] Chan W.K., Horvath T.D., Tan L., Link T., Harutyunyan K.G., Pontikos M.A., Anishkin A., Du D., Martin L.A., Yin E. (2019). Glutaminase Activity of L-Asparaginase Contributes to Durable Preclinical Activity against Acute Lymphoblastic Leukemia. Mol. Cancer Ther..

[28] Nguyen H.A., Su Y., Zhang J.Y., Antanasijevic A., Caffrey M., Schalk A.M., Liu L., Rondelli D., Oh A., Mahmud D.L. (2018). A Novel l-Asparaginase with low l-Glutaminase Coactivity Is Highly Efficacious against Both T- and B-cell Acute Lymphoblastic Leukemias In Vivo. Cancer Res..

[29] Radadiya A., Zhu W., Coricello A., Alcaro S., Richards N.G.J. (2020). Improving the Treatment of Acute Lymphoblastic Leukemia. Biochemistry.

[30] Lomelino C.L., Andring J.T., McKenna R., Kilberg M.S. (2017). Asparagine synthetase: Function, structure, and role in disease. J. Biol. Chem..

[31] Jiang J., Srivastava S., Seim G., Pavlova N.N., King B., Zou L., Zhang C., Zhong M., Feng H., Kapur R. (2019). Promoter demethylation of the asparagine synthetase gene is required for ATF4-dependent adaptation to asparagine depletion. J. Biol. Chem..

[32] Touzart A., Lengliné E., Latiri M., Belhocine M., Smith C., Thomas X., Spicuglia S., Puthier D., Pflumio F., Leguay T. (2019). Epigenetic Silencing Affects l-Asparaginase Sensitivity and Predicts Outcome in T-ALL. Clin. Cancer Res..

[33] Gwinn D.M., Lee A.G., Briones-Martin-Del-Campo M., Conn C.S., Simpson D.R., Scott A.I., Le A., Cowan T.M., Ruggero D., Sweet-Cordero E.A. (2018). Oncogenic KRAS Regulates Amino Acid Homeostasis and Asparagine Biosynthesis via ATF4 and Alters Sensitivity to L-Asparaginase. Cancer Cell.

[34] DeBlasi J.M., DeNicola G.M. (2020). Dissecting the Crosstalk between NRF2 Signaling and Metabolic Processes in Cancer. Cancers.

[35] Chiu M., Taurino G., Bianchi M.G., Kilberg M.S., Bussolati O. (2020). Asparagine Synthetase in Cancer: Beyond Acute Lymphoblastic Leukemia. Front. Oncol..

[36] Zhang B., Dong L.-W., Tan Y.-X., Zhang J., Pan Y.-F., Yang C., Li M.-H., Ding Z.-W., Liu L.-J., Jiang T.-Y. (2013). Asparagine synthetase is an independent predictor of surgical survival and a potential therapeutic target in hepatocellular carcinoma. Br. J. Cancer.

[37] Dufour E., Gay F., Aguera K., Scoazec J.-Y., Horand F., Lorenzi P.L., Godfrin Y. (2012). Pancreatic Tumor Sensitivity to Plasma L-Asparagine Starvation. Pancreas.

[38] Knott S.R.V., Wagenblast E., Khan S., Kim S.Y., Soto M., Wagner M., Turgeon M.-O., Fish L., Erard N., Gable A.L. (2018). Asparagine bioavailability governs metastasis in a model of breast cancer. Nat. Cell Biol..

[39] Aslanian A.M., Fletcher B.S., Kilberg M.S. (2001). Asparagine synthetase expression alone is sufficient to induce l-asparaginase resistance in MOLT-4 human leukaemia cells. Biochem. J..

[40] Su N., Pan Y.-X., Zhou M., Harvey R.C., Hunger S.P., Kilberg M.S. (2007). Correlation between asparaginase sensitivity and asparagine synthetase protein content, but not mRNA, in acute lymphoblastic leukemia cell lines. Pediatr. Blood Cancer.

[41] Fung M.K.L., Chan G.C.F. (2017). Drug-induced amino acid deprivation as strategy for cancer therapy. J. Hematol. Oncol..

[42] Closs E.I., Simon A., Vékony N., Rotmann A. (2004). Plasma Membrane Transporters for Arginine. J. Nutr..

[43] Kuo M.T., Savaraj N., Feun L.G. (2010). Targeted cellular metabolism for cancer chemotherapy with recombinant arginine-degrading enzymes. Oncotarget.

[44] Delage B., Luong P., Maharaj L., O’Riain C., Syed N., Crook T., Hatzimichael E., Papoudou-Bai A., Mitchell T.J., Whittaker S.J. (2012). Promoter methylation of argininosuccinate synthetase-1 sensitises lymphomas to arginine deiminase treatment, autophagy and caspase-dependent apoptosis. Cell Death Dis..

[45] Syed N., Langer J., Janczar K., Singh P.K., Nigro C.L., Lattanzio L., Coley H.M., Hatzimichael E., Bomalaski J.S., Szlosarek P.W. (2013). Epigenetic status of argininosuccinate synthetase and argininosuccinate lyase modulates autophagy and cell death in glioblastoma. Cell Death Dis..

[46] Szlosarek P.W., Grimshaw M.J., Wilbanks G.D., Hagemann T., Wilson J.L., Burke F., Stamp G., Balkwill F.R. (2007). Aberrant regulation of argininosuccinate synthetase by TNF-α in human epithelial ovarian cancer. Int. J. Cancer.

[47] Huang H.-Y., Wu W.-R., Wang Y.-H., Wang J.-W., Fang F.-M., Tsai J.-W., Li S.-H., Hung H.-C., Yu S.-C., Lan J. (2013). ASS1 as a Novel Tumor Suppressor Gene in Myxofibrosarcomas: Aberrant Loss via Epigenetic DNA Methylation Confers Aggressive Phenotypes, Negative Prognostic Impact, and Therapeutic Relevance. Clin. Cancer Res..

[48] Nicholson L.J., Smith P.R., Hiller L., Szlosarek P.W., Kimberly C., Sehouli J., Koensgen D., Mustea A., Schmid P., Crook T. (2009). Epigenetic silencing of argininosuccinate synthetase confers resistance to platinum-induced cell death but collateral sensitivity to arginine auxotrophy in ovarian cancer. Int. J. Cancer.

[49] Lan J., Tai H.-C., Lee S.-W., Chen T.-J., Huang H.-Y., Li C.-F. (2013). Deficiency in expression and epigenetic DNA Methylation of ASS1 gene in nasopharyngeal carcinoma: Negative prognostic impact and therapeutic relevance. Tumor Biol..

[50] Szlosarek P.W., Luong P., Phillips M.M., Baccarini M., Ellis S., Szyszko T., Sheaff M.T., Avril N., Stephen E. (2013). Metabolic Response to Pegylated Arginine Deiminase in Mesothelioma With Promoter Methylation of Argininosuccinate Synthetase. J. Clin. Oncol..

[51] Allen M.D., Luong P., Hudson C., Leyton J., Delage B., Ghazaly E., Cutts R., Yuan M., Syed N., Nigro C.L. (2014). Prognostic and Therapeutic Impact of Argininosuccinate Synthetase 1 Control in Bladder Cancer as Monitored Longitudinally by PET Imaging. Cancer Res..

[52] Fiedler T., Strauss M., Hering S., Redanz U., William D., Rosche Y., Classen C.F., Kreikemeyer B., Linnebacher M., Maletzki C. (2015). Arginine deprivation by arginine deiminase of Streptococcus pyogenes controls primary glioblastoma growth in vitro and in vivo. Cancer Biol. Ther..

[53] Tsai W.-B., Aiba I., Lee S.-Y., Feun L., Savaraj N., Kuo M.T. (2009). Resistance to arginine deiminase treatment in melanoma cells is associated with induced argininosuccinate synthetase expression involving c-Myc/HIF-1α/Sp4. Mol. Cancer Ther..

[54] Tsai W.-B., Long Y., Chang J.T., Savaraj N., Feun L.G., Jung M., Chen H.H.W., Kuo M.T. (2017). Chromatin remodeling system p300-HDAC2-Sin3A is involved in Arginine Starvation-Induced HIF-1α Degradation at the ASS1 promoter for ASS1 Derepression. Sci. Rep..

[55] Tsai W.-B., Long Y., Park J.-R., Chang J.T., Liu H., Rodriguez-Canales J., Savaraj N., Feun L.G., Davies M.A., Wistuba I. (2016). Gas6/Axl is the sensor of arginine-auxotrophic response in targeted chemotherapy with arginine-depleting agents. Oncogene.

[56] Tsai W.-B., Aiba I., Long Y., Lin H.-K., Feun L., Savaraj N., Kuo M.T. (2012). Activation of Ras/PI3K/ERK Pathway Induces c-Myc Stabilization to Upregulate Argininosuccinate Synthetase, Leading to Arginine Deiminase Resistance in Melanoma Cells. Cancer Res..

[57] Lin C.Y., Lovén J., Rahl P.B., Paranal R.M., Burge C.B., Bradner J.E., Lee T.I., Young R.A. (2012). Transcriptional Amplification in Tumor Cells with Elevated c-Myc. Cell.

[58] Kuo M.T., Long Y., Tsai W.B., Li Y.Y., Chen H.H.W., Feun L.G., Savaraj N. (2020). Collaboration Between RSK-EphA2 and Gas6-Axl RTK Signaling in Arginine Starvation Response That Confers Resistance to EGFR Inhibitors. Trans. Oncol..

[59] Long Y., Tsai W.-B., Chang J.T., Estécio M.R., Wangpaichitr M., Savaraj N., Feun L.G., Chen H.H., Kuo M.T. (2016). Cisplatin-induced synthetic lethality to arginine-starvation therapy by transcriptional suppression of ASS1 is regulated by DEC1, HIF-1α, and c-Myc transcription network and is independent of ASS1 promoter DNA methylation. Oncotarget.

[60] Phillips M.M., Sheaff M.T., Szlosarek P.W. (2013). Targeting Arginine-Dependent Cancers with Arginine-Degrading Enzymes: Opportunities and Challenges. Cancer Res. Treat..

[61] Zhou S., Allard P.-M., Wolfrum C., Ke C., Tang C., Ye Y., Wolfender J.-L. (2019). Identification of chemotypes in bitter melon by metabolomics: A plant with potential benefit for management of diabetes in traditional Chinese medicine. Metabolomics.

[62] Abou-Alfa G.K., Qin S., Ryoo B.-Y., Lu S.-N., Yen C.-J., Feng Y.-H., Lim H., Izzo F., Colombo M., Sarker D. (2018). Phase III randomized study of second line ADI-PEG 20 plus best supportive care versus placebo plus best supportive care in patients with advanced hepatocellular carcinoma. Ann. Oncol..

[63] Yau T.C.-C., Cheng P.N., Chan P., Chen L., Yuen J., Pang R., Fan S.T., Wheatley D.N., Poon R.T. (2015). Preliminary efficacy, safety, pharmacokinetics, pharmacodynamics and quality of life study of pegylated recombinant human arginase 1 in patients with advanced hepatocellular carcinoma. Investig. New Drugs.

[64] Mussai F., Egan S.A., Higginbotham-Jones J., Perry T., Beggs A., Odintsova E., Loke J., Pratt G., Kin P.U., Lo A. (2015). Arginine dependence of acute myeloid leukemia blast proliferation: A novel therapeutic target. Blood.

[65] Irani K., Xia Y., Zweier J.L., Sollott S.J., Der C.J., Fearon E.R., Sundaresan M., Finkel T., Goldschmidt-Clermont P.J. (1997). Mitogenic Signaling Mediated by Oxidants in Ras-Transformed Fibroblasts. Science.

[66] Abu Aboud O., Habib S.L., Trott J., Stewart B., Liang S., Chaudhari A.J., Sutcliffe J., Weiss R.H. (2017). Glutamine Addiction in Kidney Cancer Suppresses Oxidative Stress and Can Be Exploited for Real-Time Imaging. Cancer Res..

[67] Craze M.L., Cheung H., Jewa N., Coimbra N.D.M., Soria D., El-Ansari R., Aleskandarany M.A., Cheng K.W., Diez-Rodriguez M., Nolan C.C. (2017). MYC regulation of glutamine–proline regulatory axis is key in luminal B breast cancer. Br. J. Cancer.

[68] Wise D.R., DeBerardinis R.J., Mancuso A., Sayed N., Zhang X.-Y., Pfeiffer H.K., Nissim I., Daikhin E., Yudkoff M., McMahon S.B. (2008). Myc regulates a transcriptional program that stimulates mitochondrial glutaminolysis and leads to glutamine addiction. Proc. Natl. Acad. Sci. USA.

[69] Natarajan S.K., Zhu W., Liang X., Zhang L., Demers A.J., Zimmerman M.C., Simpson M.A., Becker D.F. (2012). Proline dehydrogenase is essential for proline protection against hydrogen peroxide-induced cell death. Free Radic. Biol. Med..

[70] Tołoczko-Iwaniuk N., Dziemianczyk-Pakiela D., Celińska-Janowicz K., Ościłowska I., Klupczynska A., Kokot Z., Nowaszewska K.B., Reszeć J., Borys J., Miltyk W. (2020). Proline-Dependent Induction of Apoptosis in Oral Squamous Cell Carcinoma (OSCC)—The Effect of Celecoxib. Cancers.

[71] Hancock C.N., Liu W., Alvord W.G., Phang J.M. (2016). Co-regulation of mitochondrial respiration by proline dehydrogenase/oxidase and succinate. Amino Acids.

[72] Huynh T.Y.L., Zareba I., Baszanowska W., Lewoniewska S., Pałka J. (2020). Understanding the role of key amino acids in regulation of proline dehydrogenase/proline oxidase (prodh/pox)-dependent apoptosis/autophagy as an approach to targeted cancer therapy. Mol. Cell. Biochem..

[73] Mick E., Titov D.V., Skinner O.S., Sharma R., Jourdain A., Mootha V.K. (2020). Distinct mitochondrial defects trigger the integrated stress response depending on the metabolic state of the cell. eLife.

[74] Gregory M.A., Nemkov T., Park H.J., Zaberezhnyy V., Gehrke S., Adane B., Jordan C.T., Hansen K.C., D’Alessandro A., DeGregori J. (2019). Targeting Glutamine Metabolism and Redox State for Leukemia Therapy. Clin. Cancer Res..

[75] Gwangwa M.V., Joubert A.M., Visagie M.H. (2019). Effects of glutamine deprivation on oxidative stress and cell survival in breast cell lines. Biol. Res..

[76] Shen Y.A., Hong J., Asaka R., Asaka S., Hsu F.C., Rahmanto Y.S., Jung J.G., Chen Y.W., Yen T.T., Tomaszewski A. (2020). Inhibition of the MYC-Regulated Glutaminase Metabolic Axis Is an Effective Synthetic Lethal Approach for Treating Chemoresistant Ovarian Cancers. Cancer Res..

[77] Seitz V., Butzhammer P., Hirsch B., Hecht J., Gütgemann I., Ehlers A., Lenze D., Oker E., Sommerfeld A., Von Der Wall E. (2011). Deep Sequencing of MYC DNA-Binding Sites in Burkitt Lymphoma. PLoS ONE.

[78] Yun H.R., Jo Y.H., Kim J., Shin Y., Kim S.S., Choi T.G. (2020). Roles of Autophagy in Oxidative Stress. Int. J. Mol. Sci..

[79] Li Y.-Y., Feun L., Thongkum A., Tu C.-H., Chen S.-M., Wangpaichitr M., Wu C., Kuo M.T., Savaraj N. (2017). Autophagic Mechanism in Anti-Cancer Immunity: Its Pros and Cons for Cancer Therapy. Int. J. Mol. Sci..

[80] Chen Q., Ye L., Fan J., Zhang X., Wang H., Liao S., Song P., Wang Z., Wang S., Li Y. (2017). Autophagy suppression potentiates the anti-glioblastoma effect of asparaginase in vitro and in vivo. Oncotarget.

[81] Scherz-Shouval R., Shvets E., Fass E., Shorer H., Gil L., Elazar Z. (2007). Reactive oxygen species are essential for autophagy and specifically regulate the activity of Atg. EMBO J..

[82] Ji Y., Li L., Tao Q., Zhang X., Luan J., Zhao S., Liu H., Ju D. (2017). Deprivation of asparagine triggers cytoprotective autophagy in laryngeal squamous cell carcinoma. Appl. Microbiol. Biotechnol..

[83] Changou C.A., Chen Y.-R., Xing L., Yen Y., Chuang F.Y.S., Cheng R.H., Bold R.J., Ann D.K., Kung H.-J. (2014). Arginine starvation-associated atypical cellular death involves mitochondrial dysfunction, nuclear DNA leakage, and chromatin autophagy. Proc. Natl. Acad. Sci. USA.

[84] Sosman J.A., Kim K.B., Schuchter L., Gonzalez R., Pavlick A.C., Weber J.S., McArthur G.A., Hutson T.E., Moschos S.J., Flaherty K.T. (2012). Survival in BRAF V600–Mutant Advanced Melanoma Treated with Vemurafenib. N. Engl. J. Med..

[85] Hauschild A., Grob J.-J., Demidov L.V., Jouary T., Gutzmer R., Millward M., Rutkowski P., Blank C.U., Miller W.H., Kaempgen E. (2012). Dabrafenib in BRAF-mutated metastatic melanoma: A multicentre, open-label, phase 3 randomised controlled trial. Lancet.

[86] Li Y.-Y., Wu C., Chen S.-M., Shah S.S., Wangpaichitr M., Feun L.G., Kuo M.T., Suarez M., Prince J., Savaraj N. (2016). BRAF inhibitor resistance enhances vulnerability to arginine deprivation in melanoma. Oncotarget.

[87] Li Y.-Y., Wu C., Shah S.S., Chen S.-M., Wangpaichitr M., Kuo M.T., Feun L.G.L.G., Han X., Suarez M., Prince J. (2017). Degradation of AMPK-α1 sensitizes BRAF inhibitor-resistant melanoma cells to arginine deprivation. Mol. Oncol..

[88] Brashears C.B., Barlin M., Ehrhardt W.R., Rathore R., Schultze M., Tzeng S.-C., Van Tine B.A., Held J.M. (2020). Systems level profiling of arginine starvation reveals MYC and ERK adaptive metabolic reprogramming. Cell Death Dis..

[89] Tiziani S., Kang Y., Harjanto R., Axelrod J., Piermarocchi C., Roberts W., Paternostro G. (2013). Metabolomics of the Tumor Microenvironment in Pediatric Acute Lymphoblastic Leukemia. PLoS ONE.

[90] Gaglio D., Bonanomi M., Valtorta S., Bharat R., Ripamonti M., Conte F., Fiscon G., Righi N., Napodano E., Papa F. (2020). Disruption of redox homeostasis for combinatorial drug efficacy in K-Ras tumors as revealed by metabolic connectivity profiling. Cancer Metab..

[91] Sahu N., Cruz D.D., Gao M., Sandoval W., Haverty P.M., Liu J., Stephan J.-P., Haley B., Classon M., Hatzivassiliou G. (2016). Proline Starvation Induces Unresolved ER Stress and Hinders mTORC1-Dependent Tumorigenesis. Cell Metab..

[92] Klapproth K., Wirth T. (2010). Advances in the understanding of MYC-induced lymphomagenesis. Br. J. Haematol..

[93] Marchingo J.M., Sinclair L.V., Howden A.J., Cantrell D.A. (2020). Quantitative analysis of how Myc controls T cell proteomes and metabolic pathways during T cell activation. eLife.

[94] Van Geldermalsen M., Wang Q., Nagarajah R., Marshall A.D., Thoeng A., Gao D., Ritchie W., Feng Y., Bailey C.G., Deng N. (2016). ASCT2/SLC1A5 controls glutamine uptake and tumour growth in triple-negative basal-like breast cancer. Oncogene.

[95] Kuo M.T., Tsai W.-B., Wangpaichitr M., Tsukamoto T., Savaraj N., Feun L.G., Kuo M.T. (2013). Arginine Deiminase Resistance in Melanoma Cells Is Associated with Metabolic Reprogramming, Glucose Dependence, and Glutamine Addiction. Mol. Cancer Ther..

[96] Le A.-P., Zhang L.-L., Liu W., Shi Y.-F. (2016). Cantharidin inhibits cell proliferation and induces apoptosis through G2/M phase cell cycle arrest in hepatocellular carcinoma stem cells. Oncol. Rep..

[97] Tameire F., Verginadis I.I., Leli N.M., Polte C., Conn C.S., Ojha R., Salinas C.S., Chinga F., Monroy A.M., Fu W. (2019). ATF4 couples MYC-dependent translational activity to bioenergetic demands during tumour progression. Nat. Cell Biol..

[98] Xu X., Watt D.S., Liu C. (2015). Multifaceted roles for thymine DNA glycosylase in embryonic development and human carcinogenesis. Acta Biochim. Biophys. Sin..

[99] Bott A.J., Peng I.-C., Fan Y., Faubert B., Zhao L., Li J., Neidler S., Sun Y., Jaber N., Krokowski D. (2015). Oncogenic Myc Induces Expression of Glutamine Synthetase through Promoter Demethylation. Cell Metab..

[100] Gao P., Tchernyshyov I., Chang T.-C., Lee Y.-S., Kita K., Ochi T., Zeller K.I., De Marzo A.M., Van Eyk J.E., Mendell J.T. (2009). c-Myc suppression of miR-23a/b enhances mitochondrial glutaminase expression and glutamine metabolism. Nat. Cell Biol..

[101] Momcilovic M., Bailey S.T., Lee J.T., Fishbein M.C., Braas D., Go J., Graeber T.G., Parlati F., Demo S., Li R. (2018). The GSK3 Signaling Axis Regulates Adaptive Glutamine Metabolism in Lung Squamous Cell Carcinoma. Cancer Cell.

[102] Liu W., Le A., Hancock C., Lane A.N., Dang C.V., Fan T.W.-M., Phang J.M. (2012). Reprogramming of proline and glutamine metabolism contributes to the proliferative and metabolic responses regulated by oncogenic transcription factor c-MYC. Proc. Natl. Acad. Sci. USA.

[103] Kardos G.R., Wastyk H.C., Robertson G.P. (2015). Disruption of Proline Synthesis in Melanoma Inhibits Protein Production Mediated by the GCN2 Pathway. Mol. Cancer Res..

[104] Ren W., Li Y., Xia X., Guo W., Zhai T., Jin Y., Che Y., Gao H., Duan X., Ma H. (2018). Arginine inhibits the malignant transformation induced by interferon-gamma through the NF-kappaB-GCN2/eIF2alpha signaling pathway in mammary epithelial cells in vitro and in vivo. Exp. Cell Res..

[105] Wortel I.M.N., van der Meer L.T., Kilberg M.S., van Leeuwen F.N. (2017). Surviving Stress: Modulation of ATF4-Mediated Stress Responses in Normal and Malignant Cells. Trends Endocrinol. Metab..

[106] Linares J.F., Cordes T., Duran A., Reina-Campos M., Valencia T., Ahn C.S., Castilla E.A., Moscat J., Metallo C.M., Diaz-Meco M.T. (2017). ATF4-Induced Metabolic Reprograming Is a Synthetic Vulnerability of the p62-Deficient Tumor Stroma. Cell Metab..

[107] Cherasse Y., Maurin A.-C., Chaveroux C., Jousse C., Carraro V., Parry L., Deval C., Chambon C., Fafournoux P., Bruhat A. (2007). The p300/CBP-associated factor (PCAF) is a cofactor of ATF4 for amino acid-regulated transcription of CHOP. Nucleic Acids Res..

[108] Long Y., Tsai W.-B., Wang D., Hawke D., Savaraj N., Feun L.G., Hung M.-C., Chen H.H., Kuo M.T. (2017). Argininosuccinate synthetase 1 (ASS1) is a common metabolic marker of chemosensitivity for targeted arginine- and glutamine-starvation therapy. Cancer Lett..

[109] Xiang Y., Stine Z.E., Xia J., Lu Y., O’Connor R.S., Altman B.J., Hsieh A.L., Gouw A.M., Thomas A.G., Gao P. (2015). Targeted inhibition of tumor-specific glutaminase diminishes cell-autonomous tumorigenesis. J. Clin. Investig..

[110] Pavlova N.N., Hui S., Ghergurovich J.M., Fan J., Intlekofer A.M., White R.M., Rabinowitz J.D., Thompson C.B., Zhang J. (2018). As Extracellular Glutamine Levels Decline, Asparagine Becomes an Essential Amino Acid. Cell Metab..

[111] Lowman X.H., Hanse E.A., Yang Y., Gabra M.B.I., Tran T.Q., Li H., Kong M. (2019). p53 Promotes Cancer Cell Adaptation to Glutamine Deprivation by Upregulating Slc7a3 to Increase Arginine Uptake. Cell Rep..

[112] Bröer S. (2020). Amino Acid Transporters as Targets for Cancer Therapy: Why, Where, When, and How. Int. J. Mol. Sci..

[113] Bröer S. (2013). The SLC38 family of sodium–amino acid co-transporters. Pflügers Archiv..

[114] Bolzoni M., Chiu M., Accardi F., Vescovini R., Airoldi I., Storti P., Todoerti K., Agnelli L., Missale G., Andreoli R. (2016). Dependence on glutamine uptake and glutamine addiction characterize myeloma cells: A new attractive target. Blood.

[115] Bröer A., Rahimi F., Bröer S. (2016). Deletion of Amino Acid Transporter ASCT2 (SLC1A5) Reveals an Essential Role for Transporters SNAT1 (SLC38A1) and SNAT2 (SLC38A2) to Sustain Glutaminolysis in Cancer Cells. J. Biol. Chem..

[116] Lopez A.B., Wang C., Huang C.C., Yaman I., Li Y., Chakravarty K., Johnson P.F., Chiang C.-M., Snider M.D., Wek R.C. (2007). A feedback transcriptional mechanism controls the level of the arginine/lysine transporter cat-1 during amino acid starvation. Biochem. J..

[117] Krall A.S., Xu S., Graeber T.G., Braas D., Christofk H.R. (2016). Asparagine promotes cancer cell proliferation through use as an amino acid exchange factor. Nat. Commun..

[118] Biancur D.E., Paulo J.A., Małachowska B., Del Rey M.Q., Sousa C.M., Wang X., Sohn A.S.W., Chu G.C., Gygi S.P., Harper J.W. (2017). Compensatory metabolic networks in pancreatic cancers upon perturbation of glutamine metabolism. Nat. Commun..

[119] Hall P.E., Lewis R., Syed N., Shaffer R., Evanson J., Ellis S., Williams M., Feng X., Johnston A., Thomson J. (2019). A Phase I Study of Pegylated Arginine Deiminase (Pegargiminase), Cisplatin, and Pemetrexed in Argininosuccinate Synthetase 1-Deficient Recurrent High-grade Glioma. Clin. Cancer Res..

[120] Yang T.-S., Lu S.-N., Chao Y., Sheen I.-S., Lin C.-C., Wang T.-E., Chen S.-C., Wang J.-H., Liao L.-Y., Thomson J. (2010). A randomised phase II study of pegylated arginine deiminase (ADI-PEG 20) in Asian advanced hepatocellular carcinoma patients. Br. J. Cancer.

[121] Hijiya N., Van Der Sluis I. (2015). Asparaginase-associated toxicity in children with acute lymphoblastic leukemia. Leuk. Lymphoma.

[122] Cecconello D.K., De Magalhães M.R., Werlang I.C.R., Lee M.L.D.M., Michalowski M.B., Daudt L.E. (2020). Asparaginase: An old drug with new questions. Hematol. Transfus. Cell Ther..

[123] Ko R.H., Jones T.L., Radvinsky D., Robison N., Gaynon P.S., Panosyan E.H., Avramis I.A., Avramis V.I., Rubin J., Ettinger L.J. (2015). Allergic reactions and antiasparaginase antibodies in children with high-risk acute lymphoblastic leukemia: A children’s oncology group report. Cancer.

[124] Pession A., Valsecchi M.G., Masera G., Kamps W.A., Magyarosy E., Rizzari C., Van Wering E.R., Nigro L.L., Van Der Does A., Locatelli F. (2005). Long-Term Results of a Randomized Trial on Extended Use of High Dose l-Asparaginase for Standard Risk Childhood Acute Lymphoblastic Leukemia. J. Clin. Oncol..

[125] Amylon M.D., Shuster J., Pullen J., Berard C., Link M.P., Wharam M., Katz J., Yu A., Laver J., Ravindranath Y. (1999). Intensive high-dose asparaginase consolidation improves survival for pediatric patients with T cell acute lymphoblastic leukemia and advanced stage lymphoblastic lymphoma: A Pediatric Oncology Group study. Leukemia.

[126] Bourgeois W. (2020). 50 Years Ago in The Journal of Pediatrics: L-Asparaginase Therapy in Pediatric Acute Lymphoblastic Leukemia: Optimizing Efficacy and Minimizing Toxicity. J. Pediatr..

[127] Zimmermann S.C., Duvall B., Tsukamoto T. (2019). Recent Progress in the Discovery of Allosteric Inhibitors of Kidney-Type Glutaminase. J. Med. Chem..

[128] Huang Q., Stalnecker C., Zhang C., McDermott L.A., Iyer P., O’Neill J., Reimer S., Cerione R.A., Katt W.P. (2018). Characterization of the interactions of potent allosteric inhibitors with glutaminase C, a key enzyme in cancer cell glutamine metabolism. J. Biol. Chem..

[129] D’Aniello C., Patriarca E.J., Phang J.M., Minchiotti G. (2020). Proline Metabolism in Tumor Growth and Metastatic Progression. Front. Oncol..

[130] Ding J., Kuo M.-L., Su L., Xue L., Luh F., Zhang H., Wang J., Lin T.G., Zhang K., Chu P. (2017). Human mitochondrial pyrroline-5-carboxylate reductase 1 promotes invasiveness and impacts survival in breast cancers. Carcinogenesis.

